# Perceptions of School Management on the Relationship between School Nutrition and Development of Non-Communicable Diseases in a Rural South African District: A Qualitative Study

**DOI:** 10.3390/ijerph19010432

**Published:** 2021-12-31

**Authors:** Sibusiso Cyprian Nomatshila, Teke Ruffin Apalata, Sikhumbuzo A. Mabunda

**Affiliations:** 1 Department of Public Health, Faculty of Health Sciences, Walter Sisulu University, Mthatha 5117, South Africa; 2 Department of Laboratory Medicine, Faculty of Health Sciences, Walter Sisulu University, Mthatha 5117, South Africa; tapalata@wsu.ac.za; 3 The George Institute for Global Health, University of New South Wales, Sydney 2042, Australia; smabunda@georgeinstitute.org.au

**Keywords:** nutrition program, vendor system, qualitative study, obesity, non-communicable diseases

## Abstract

Globally, noncommunicable diseases (NCDs) were responsible for 41 million deaths in 2016, with the majority of these occurring in low- and middle-income countries. These diseases are on the rise as a result of unhealthy, low-quality, and unbalanced diets, which have resulted in overweight and obesity. The National School Nutrition Program (NSNP) was created to regulate the foods sold to schoolchildren. The aim of this study was to ascertain school management teams’ perspectives on the relationship between the NSNP and the development of lifestyle diseases. A phenomenological qualitative study using focus group discussions among 16 purposively selected members of the school management teams were conducted in Mt Frere, Eastern Cape in 2016. The narrative data were analyzed using Tesch’s eight-phase thematic analysis approach. The data analysis revealed two themes (NSNP and the vendor system) and six sub-themes. The NSNP was viewed as making a significant contribution to children’s food security, thereby improving academic output. However, reengineering of the NSNP was needed through improved budgeting and inclusion of breakfast in the menu to control NCDs risk factors. The current implementation of the vendor system did not support reduction of NCDs risk factors. Improved implementation of the guide to the vendor system is needed.

## 1. Introduction

In 2018, the World Health Organization (WHO) reported that non-communicable diseases (NCDs) accounted for around 41 million (or 71%) of all worldwide deaths, of which 77% came from low- and middle-income countries (LMICs) [1]. About 85% of these deaths were premature deaths [1]. This represented a 3% increase over four years from 2014, with NCDs accounting for 38 million of all global deaths, with most (68%) of these deaths occurring in LMICs [2,3]. Non-communicable diseases such as cardiovascular diseases, diabetes, some respiratory diseases, and cancers are reported to have the potential of increasing exponentially by the year 2040, especially in LMICs, and will account for many premature deaths [4]. 

Non-communicable diseases are on the rise because of a variety of factors, including unhealthy, low-quality diets; excessive and unbalanced intake leads to overweight and obesity, both of which are risk factors for NCDs [5]. Diet-related NCDs impose a significant burden on populations, families, and the healthcare system [6]. Unhealthy diets, poor quality diets, and malnutrition are reported as the major risk factor for NCDs and have been attributed to dramatic global changes in food production, marketing, supply, and consumption [5].

The National School Nutrition Program (NSNP) policy was developed by the Department of Basic Education in South Africa for implementation by all public schools in order to assist in providing proper nutrition and improving the children’s wellbeing through guidance on the structure of the lunch box, foods to be served at schools, guidelines on the establishment of tuck-shops at school, what these tuck-shops should sell to children and teachers, and the establishment of school gardens [7]. In a study conducted in 2015 by Gresse and colleagues in South Africa, it transpired that there was a challenge with the management of tuck-shops arising from the fact that they did not form part of the school system and were not accountable to the same structures as schools [8]. As such, adequate training for their managers is of paramount importance. 

School management teams (SMTs) are empowered by the South African Schools Act (SASA) (1996) with the responsibility of governance to schools whilst they oversee the implementation of a successful NSNP through promotion of the availability of healthy food alternatives from school tuck shops/vendors on school premises [7,9]. Challenges in the management of tuck-shops and the general vendor system in schools as part of the NSNP expose school children to unregulated sale of low cost and prohibited food items with no nutritional advantage [10], thus weakening controls of NCDs risk factors. These weakened controls are exacerbated by the flaws in the functioning of the NSNP: inconsistent food distribution, decayed and poor-quality food, limited variation, and with no fruit or vegetables [11,12]. This situation defeats the purpose of the establishment of the NSNP, resulting in children having to carry money to school in order to obtain alternate meals or snacks that are often high in fat content and calories as part of their daily diets [13]. A study conducted in Nepal revealed poor coordination among relevant stakeholders which was identified as a barrier to the success of school nutrition programs [14]. Shrestha et al. proposed better coordination and collaboration to continue efforts to implement and strengthen the program. The purpose of this study was to ascertain school management teams’ perceptions of the relationship between the school nutrition program and the development of NCDs. 

## 2. Materials and Methods

### 2.1. Study Design 

This phenomenological qualitative study used focus group discussions to understand the perceptions of school governance leadership about the implementation of the National School Nutrition Program and its effects on the development of NCDs.

### 2.2. Setting 

This study was conducted in Mt Frere, a rural town in the north-western part of the Alfred Nzo District Municipality. Most of the population resides in the rural areas surrounding the town [15]. The current study was carried out within Mt Frere schools. Mt Frere has 217 schools, with this study taking place in two of them, a primary and a secondary school.

### 2.3. Population

Members of school management teams including two principals, one deputy principal, three department heads, eight parent representatives, and two educators took part in the study. These participants were seen as having positions of authority in schools and were thought to be critical for the focus group discussions (FGDs). Each focus group had 6–10 participants. Even though these participants did not represent the general population of school management teams in schools throughout the Eastern Cape, they did represent school management teams in Mt Frere. 

### 2.4. Sampling Procedure 

Purposive sampling was used to select participants for the FGDs on selected days for data collection. Each focus group discussion had 6–10 participants, all of whom were members of the school management, to explore their perceptions on the associations between the School Nutrition Program and the development of NCDs in Mt Frere. The population of interest for this study, which is SMTs in schools where children of the Prospective Urban and Rural Epidemiology study (PURE) families studied, was found in 25 of the 217 schools, with 5 (20%) of these schools being sampled through simple random sampling. However, SMTs, which included educators and parent representatives, were obtained from only 2 schools, owing to data saturation. 

### 2.5. Data Collection 

#### Focus Group Discussions 

All participants were given the opportunity to take part in focus group discussions (FGDs) in exploring the perceptions of school administration’s on NCDs and what could be done to prevent them. Two focus group discussions (FGDs) were held, each lasting a minimum of 60 min. An expert in the field of public health and health promotion moderated the discussion. An interview guide was used to ask open-ended questions. All focus group discussions were held in IsiXhosa, a widely spoken local language in the area. Discussions were audio-recorded and then meticulously transcribed for analysis by a skilled transcriber. Table 1 shows main questions asked during FGDs. 

### 2.6. Data Analyses

Descriptive data from the FGDs were analyzed immediately after FGDs using the Tesch’s eight phases thematic analysis approach [16]. Permission was sought from the participants to voice-record the discussions of FGDs. All data collected were precisely transcribed from the sound recorder to avoid misinterpretation of useful information. The transcriptions were done from vernacular (IsiXhosa) into English and back to IsiXhosa to avoid distortion. The audio recorder was run a number of times while being stopped intermittently to accurately capture substance in the recordings. The field notes were also typed, and their contents infused into the main transcripts for data comprehensiveness. Furthermore, there was peer checking of both transcripts by the first (Sibusiso Cyprian Nomatshila) and third au-thor (Sikhumbuzo A. Mabunda) to further ensure consistency of the final transcripts with the audio recordings. Themes and sub-themes were developed from categories with similar meanings; an independent coder was used to confirm the themes. A test-retest coding and clustering approach was used. All sound recordings were thereafter destroyed to maintain confidentiality principles. 

### 2.7. Ethics and Consent 

The study was approved by the Walter Sisulu University’s Human Research Ethics and Biosafety Committee for ethical approval (070/15). Permission to conduct the study in schools was also sought from the Eastern Cape province’s Departments of Health and Basic Education. Participants provided written informed consent with full details about the study and reporting channels. All research records were kept in a locked filing system, and all electronic data were coded and secured with a password-protected file. Participants’ identities were not recorded or disclosed in any way.

### 2.8. Trustworthiness

This relates to the trustworthiness of the qualitative research study. Credibility, confirmability, transferability, and dependability in the study were implemented in line with Lincon and colleagues’ model [17]. This was accomplished by increasing the amount of time spent with participants to ensure consistency and a better understanding of their responses to NCD risk factors. Respondents were encouraged to take ownership of the research as a means of improving their knowledge and skills in preventing NCD risk factors. The use of tape recording and note-taking ensured that a wide range of perspectives and information was collected and aligned to ensure accuracy in recording responses.

## 3. Results

The data for this study were collected from two FGDs with a total of 16 participants from school management teams in Mt Frere, Eastern Cape province. There were 10 participants in the first FGD which lasted for 60 min whilst the second FGD had 6 participants and took 70 min to complete. 

Table 2 shows two main themes and six sub-themes that emerged from the data.

### 3.1. Theme 1: National School Nutrition Program (NSNP)

#### 3.1.1. Subtheme 1: Poverty 

The implementation of the NSNP, policy framework and implementation, support system, and compliance were expressed as key areas in the school system. Participants expressed their appreciation of the NSNP and indicated that it was a good initiative that contributed meaningfully towards alleviation of poverty and in improving academic performance among learners.

“*It is good for the case of children in our areas because government came and looked at quintiles which have parents who are not working and under poverty*”.

Participant 1, FGD 1

The introduction of the NSNP was commended in that it provided a long-needed approach to enhancing school performance. 

#### 3.1.2. Subtheme 2: School Attendance

“*It is good because children know that they surely will have something to eat at school and that improves school attendance*”.

Participant 1, FGD 2

Educators felt that the introduction of the NSNP with its standard rule of serving the meals at least before 10 a.m. provided a chance of mediating risks of skipping breakfast for children from these communities in Mt Frere. More could be done in building diets and meal systems that could yield better health outcomes for their students if school management was given the opportunity to use their ideas as school leaders and ensured the execution of the policy in a fashion that suited their local situations. 

#### 3.1.3. Subtheme 3: Menu Budgeting 

One of the participants suggested that,

“*… if government could help with including a boiled egg, a slice of bread and a glass of milk*”.

Participant 2, FGD 1

This sort of adjustment would require radical financial support which was seen as deficient by the participants regardless of the value it could bring in bridging the gap caused by skipping breakfast. This was supported by another participant:

“*menu needs to be improved and budget be increased*”.

Participant 3, FGD 2

The NSNP listed the general rules on its implementation and highlighted the desired menu which promoted the use of locally preferred foods. It does not stipulate a strict rule that no variations or substitutions on any given day are permitted. 

Participants expressed their desire for the proper implementation of the NSNP but felt that the implementation of the policy became difficult because of the budget provided for it. One participant indicated barriers to the implementation of the NSNP as,

“*it will be difficult for the proper implementation with small budget. The vision is high but budget is low*”.

Participant 2, FGD 2

Participants expressed concerns in the budgetary constraints which were exposing their children to different forms of risks and compromise. One participant suggested that the budgetary barriers were considered as exposing the schools to a compromised quality of foods and said,

“*budget prevents proper implementation of the NSNP and we end up buying cheaper foods thus compromising quality and often look for specials which often have close to expiring foods*”.

Participant 4, FGD 1

This was also echoed in the other FGD where participants expressed the view that the situation was exacerbated by the poor food preparation skills among food handlers. 

#### 3.1.4. Subtheme 4: Food Handling and Preparations 

Participants felt that on top of the compromised quality of food they bought, food was also not prepared in a palatable manner due to lack of skills by those who prepared it.

“*Training of food handlers is needed as preparing meals for a thousand children requires a skill and without a skill food won’t be tasty and children won’t eat it leading to high food wastages*”.

Participant 5, FGD 2

### 3.2. Theme 2: Vendor System

#### Subtheme 1: Sold Items 

Based on the quality of food prepared at school and its palatability, participants indicated that children find themselves having to buy something from the vendors who often do not sell the prescribed items in accordance with the guide for tuck-shops. Participants reported that they had attempted to guide the vendors on what needed to be sold but compliance was very low as they reported that children liked the stuff that was sold to them. One participant expressed that,

“*We are aware of dangers of chips and we have started to discuss with vendors to stop the selling of these including sweets. We have asked them to sell healthy stuff but we have trouble with compliance*”.

Participant 5, FGD 2

The school children eat sweets, fat cakes (fried in oil), chips, and dozens of unhealthy items that are prohibited in terms of the NSNP guide. It was reported that compliance by the vendors was challenged by “soft rules” on what was acceptable inside school premises and what was not. Schools could not enforce anything without a written school policy and support of the district office. Failure to ensure compliance was in one case put to the test: learners who were found with prohibited food items inside school premises were requested to bring a parent to school the following day; however, the parents ignored this. There was also the concept of informing parents that performance reports were going to be withheld until they came to the school but that was also not supported and was opposed by the district office. The participants felt that the district office was not providing adequate support to the prevention of the risk factors and their children were at risk in turn. One participant reported,

“*we would have a problem with the district that would call and say release that report to the parent immediately*”.

Participant 7, FGD 2

It appeared to be common that schools were not at liberty to develop and apply rules in line with the situations they found themselves in. One participant felt that they were not permitted by the district office to be innovative and to apply their minds. As such, they were always at odds with the district office and ended up letting things go just for the sake of peace. 

## 4. Discussion

This study assessed the perceptions of school management teams on the association between school nutrition programs and development of non-communicable diseases among learners. Participants in the study felt that the National School Nutrition Program (NSNP) contributed immensely towards changing the situation in their communities and schools as they had needy children. This is supported by Bartfeld and Ahn [18] who reported that the provision of school nutrition programs contributed meaningfully in situations of food insecurity, leading to children worrying less about food and focusing on educational outcomes. 

According to the findings of the current study, the NSNP had a major role in reducing poverty in society by providing nutritious food to children from low-income families. Such healthy nutrition provided by NSNP has been commended for ensuring that children were kept at school with significantly lowered rates of absenteeism [19]. Improved school attendance, together with improved performance, was considered a perfect recipe for ensuring improved literacy levels in a society, thus leading to improved employment opportunities for the near future. Poverty and food insecurity were found to be major contributors to high rates of school dropout and poor academic performance, even among those who remained in school. Food insecurity has been linked to a number of irreversible educational and developmental outcomes [20,21]. The consequences of food insecurity have been shown to impair children’s behavior and emotional well-being. However, this study discovered that children’s school attendance was boosted by their assurance that they would get something to eat at school. This concept of school nutrition was not only a South African phenomenon, but also a global phenomenon. To address the food insecurity gap, the US Department of Agriculture [22] expressed support for the establishment of a healthy nutrition environment in school. The US Department of Agriculture acknowledged that there were many factors that played a role in the development of childhood obesity and, as such, school nutrition programs could play a role in combating childhood obesity.

According to the findings of this study, the South African NSNP needs to be reengineered and budgeted better in order to include breakfast in the form of a boiled egg and a glass of milk. Breakfast is said to be important in preventing children from being inactive in class, collapsing in class due to hunger, and performing poorly in school. This suggestion of reengineering of the NSNP to include breakfast was supported by a study conducted in South Africa in 2015 [23]. Addition of breakfast in the school nutrition program would reduce the effects of skipping breakfast, which is a risk factor for non-communicable diseases such as cardiovascular diseases and diabetes [24]. There is evidence suggesting that consumption of breakfast contributes to a reduced body mass index and ultimately reduces chances of overweight among children and adolescents [25]. These findings were supported by Kesztyüs et al. [26] who reported that there was an association between skipping breakfast and abdominal obesity among children in lower grades at school. Kesztyüs and colleagues suggested that schools need to offer breakfast before the school day begins on a regular basis. In a study conducted in South Africa in 2012, it was established that there was a strong perception that improved concentration and classroom interactions were strongly associated with breakfast programs in a studied population [27].

Introduction of breakfast would mean that the design of the school nutrition program should be restructured. This study found that the menus at schools were reported as not being helpful to the school management team in addressing nutritional needs and academic impact. According to the participants, this was due to the imposed and inflexible approach of the district education department through nutrition coordinators who did not accommodate local situations. According to the findings of this study, schools were obligated to adopt the menu as described in the NSNP despite having restricted funds. This was said to have resulted in the purchase of foods on special offers that were, in most cases, of poor quality. That imposed approach to the menu also did not take into account locally desired and used foods. 

The menu in the NSNP according to DBE [7] reflected that,

“School menus should offer tasty and adequate meals which must fulfil at least 30% of the daily nutritional needs of learners per meal. It is important to serve a balanced meal which is composed of:

- Protein:
- vegetable protein e.g., Soya products, dried beans, lentils, nuts and dried peas or
- animal protein e.g., meat, milk, eggs and fish (depending on affordability)
- Starch: e.g., maize meal, samp, mealie rice, rice, bread, potatoes
- Vegetables: at least one green and one red or yellow or orange vegetable per meal” [7].

In line with the provisions of the NSNP, the school menu as prescribed by the district specified items that needed to be adhered to. Participants expressed that flexibility was not provided by the district in the local implementation of the policy or menu. In this sense, the menu as provided by the district was not properly consistent with the provisions of the NSNP. 

The NSPN further recorded,

“Very important

- Selected menus should be socially acceptable.
- Use of indigenous food in menus is encouraged.
- Specifications of new menu option inclusions are obtainable from the District/Circuit NSNP officer.

Peanut butter may only be used if quality assurance standards by the Department of Health have been met” [7].

This study also revealed that, in addition to the already tainted quality of the food supply, food preparation was also tainted as a result of unskilled food handlers. Skills development was found necessary to ensure that food was not wasted given minimal resources in schools. The NSNP requires that the food handlers be trained on health and hygiene standards. This would enhance the quality of meals prepared [7]. However, it has been stated that food handlers in the NSNP are frequently overworked; some are unemployed as a result of being hired on short-term rotating contracts; and this is aggravated by unemployability due to a lack of training [28]. This often results in poor quality of food being prepared by handlers, which was reported to be a factor leading learners turning to the vendors to buy fat cakes, chips/crisps/fries, and sweets and other foods that are NCD risk factors. These undesirable low-cost items sold by vendors pose risks of development of NCD risk factors like obesity. Open availability of the unhealthy foods from vendors encourages children to buy them while reducing their desire to consume healthy food prepared and provided by the school [29]. The findings of this study show that people were aware of harmful foods supplied to children by vendors, and that the health consequences of these foods were fully recognized. It transpired that the vendor space needed to be controlled. However, participants felt that more support from the district office was required for the successful control of the space. This support by authorities was also reported as only minimally available within nutrition education [30]. This is in line with the findings of this study that whenever a student had violated school regulations on acceptable food within school premises, parents would be required to come to school and resolve such dispute with authorities. Parents, on the other hand, do not always attend to such arguments since they perceive them to be of lesser importance. Participants revealed that schools would withhold progress reports in order to compel parents to come to school, but the district officials could call and instruct them to release the report immediately. This has been described as undermining efforts to reduce the impact of NCD risk factors in the school settings. 

The burden of NCDs can be attributed to aggressive marketing of unhealthy food, alcohol, and tobacco in LMICs, which includes the major campaigns used by manufacturers that undermine the existence of regulations and guidelines aimed at reducing behavioral risk factors for NCDs [4]. Such policies, regulations, and guidelines are essential in the controlling of what is sold, and in empowering the population on essential information required before one purchases any food product. It has been reported that whenever government administration established firmer controls to mediate progression of NCDs, the food and beverage industries tend to obstruct public policy development to maximize their gains [31,32]. 

In an effort to address such interferences by manufacturers, administrations have developed instruments to guide practice of good governance with effective systems to neutralize any potential conflicting interests [33]. Regulatory frameworks are designed to control supply and selling of foods to children, especially during an era in which the demand for and supply of meat, dairy products, sugary beverages, and processed and ultra-processed foods has increased exponentially [34]. With this awareness, the government of South Africa developed a National School Nutrition Program (NSNP) to address and mediate nutritional disparities within populations [7]. This guiding policy also regulates the establishment of outlets and vendor systems specifying what needs to be sold to children at schools. It was suggested that school nutrition programs can ensure school children have access to diversified nutrition, important for healthy growth and development [5]. Branca et al. [5] further stress that the nutrition programs could play an important role in limiting children’s exposure to unhealthy foods such as are sold in outlets and by vendors. 

## 5. Limitations 

Even though efforts were made to reduce limitations, some limitations were faced. The study was limited only to schools where children of the Prospective Urban Rural Epidemiology study (PURE) families went and not the overall population of Mt Frere. Parents from these families were eligible for being part of the school governing body and ultimately school management team. As such, it cannot be considered representative of Mt Frere. Nevertheless, the findings of the study illustrate an acceptable understanding of the perceptions on the association between school nutrition programs and development of future lifestyle diseases among learners. The study did not include all stakeholders necessary for policy development and change; however, this can be best implemented by future studies. Due to limited scope of data, the current study did not adequately determine advantages and disadvantages of the school nutrition program. The sample size of the study could not provide health and nutrition framework themes. Despite all the limitations, this study determined the role of the school nutrition program in fighting poverty among the rural poor children. This resulted in improved school attendance, reduction in drop-out rates, and improved throughput. This study remained valuable in creating a body of knowledge especially on school nutrition programs in rural communities of South Africa.

## 6. Conclusions

Though the NSNP has made significant contributions towards the fight against poverty and the improvement of academic outcomes, the control of NCD risk factors posed by its poor implementation and insufficient government support remains a challenge in Mt. Frere’s rural schools. The vendor system in schools is the major weak link in the implementation of the NSNP and hosts risks for the future development of NCDs in communities. The NSNP, as an enabling factor towards good nutrition and control of NCD risk factors in school settings, is challenged by its poor implementation, resulting in the compromised health of students in school settings. Enabling people to take control of health determinants would necessitate the development and implementation of enabling factors such as policy in this situation and commitment from affected individuals. To improve the NSNP’s effectiveness and viability, school authorities must commit to reengineering it at the local level. This process would then take a long time to implement because numerous stakeholders and policy actors would need to be thoroughly consulted. However, the centralization of authority at district office level seems to hinder rather than enhance performance at school level. This suggests that the fight against those NCDs risk factors is going to be a long one. Radical decisions need to be made on how to control vendor space and ensure compliance in the implementation of the guide to vendors. Future studies should consider inclusive implementation research studies where stakeholders necessary to change the policy in school programs or vendor systems are included. Such stakeholders could include school governing bodies and sub-district, district, and provincial education and health departments. A study involving the broader population is needed for the development of a nutrition and health promotion framework to mitigate the extent of the development of lifestyle diseases. 

## Figures and Tables

**Table 1:** Focus group discussions questions.

Subject	Question
Health issues	What do you understand about NCDs?
	What are your perceptions on risks of foods sold to children at school?
	In your views, what is the effect of a school learner’s diet on the development of NCDs later on in life?
Policies, National School Nutrition Program, and vendors	What are your perceptions of NSNP?
	Which areas in the implementation of NSNP do you think require improvement?
	What is the role of each stakeholder in its implementation?
	What is your perception on the barriers to the implementation of NSNP?
	How can the vendor system be controlled/managed?
	What do you think can be done to control food items that access school premises?

**Table 2:** Themes and sub-themes.

Theme	Subthemes
National School Nutrition Program	1.1 Poverty
	1.2 School Attendance
	1.3 Menu Budgeting
	1.4 Food Handling and Preparations
2. Vendors’ system	2.1 Sold Items
	2.2 District Support

## Data Availability

Data are available on request though it will be guided by research regulations, POPIA, and the confidentiality agreement with participants.
